# Utilization of and Attitudes towards Traditional Chinese Medicine Therapies in a Chinese Cancer Hospital: A Survey of Patients and Physicians

**DOI:** 10.1155/2012/504507

**Published:** 2012-10-11

**Authors:** Jennifer L. McQuade, ZhiQiang Meng, Zhen Chen, Qi Wei, Ying Zhang, WenYing Bei, J. Lynn Palmer, Lorenzo Cohen

**Affiliations:** ^1^Department of Internal Medicine, Hospital of the University of Pennsylvania, 3400 Spruce Street, Philadelphia, PA 19104, USA; ^2^Department of Integrative Oncology, Fudan University Shanghai Cancer Center, 270 Dongan Road, Shanghai 200032, China; ^3^Department of Palliative Care and Rehabilitation Medicine, The University of Texas MD Anderson Cancer Center, 1515 Holcombe Boulevard, Unit 0462, Houston, TX 77030, USA; ^4^Department of Behavioral Science, The University of Texas MD Anderson Cancer Center, 1515 Holcombe Boulevard, Unit 0462, Houston, TX 77030, USA

## Abstract

*Background*. In China, the use of traditional Chinese medicine (TCM) is very popular, but little is known about how it is integrated with conventional cancer care. We conducted parallel surveys of patients and physicians on TCM utilization. *Methods*. Two hundred forty-five patients and 72 allopathic physicians at the Fudan University Shanghai Cancer Center completed questions on their use of and attitude towards TCM. *Results*. Patient mean age was 51, with 60% female. Eighty-three percent of patients had used TCM. Use was greatest for Chinese herbal medicine (CHM; 55.8%). Only 1.3% of patients used acupuncture and 6.8% Qi Gong or Tai Qi. Sixty-three percent of patients notified their oncologist about TCM use. The most common reason for use was to improve immune function. CHM was often used with a goal of treating cancer (66.4%), a use that 57% of physicians agreed with. Physicians were most concerned with interference with treatment, lack of evidence, and safety. Ninety percent of physicians have prescribed herbs and 87.5% have used TCM themselves. *Conclusion*. The use of TCM by Chinese cancer patients is exceptionally high, and physicians are generally well informed and supportive of patients' use. Botanical agents are much more commonly used than acupuncture or movement-based therapies.

## 1. Introduction

Complementary and alternative medicine (CAM) is defined as a group of diverse medical and health care systems, practices, and products that are not generally considered part of conventional medicine [1]. Worldwide, use of CAM among cancer patients varies widely [2], ranging from 7% to 64% globally and averaging 40% in western countries [3]. 

The definition of CAM is culturally bound. In China, CAM is largely dominated by traditional Chinese medicine (TCM). TCM is a distinct medical system that has evolved in China over a period of 5000 years. TCM refers to the theory underlying this system and to its most common therapeutic methods: acupuncture, herbology, food therapy, Tui Na (Chinese acupressure and massage), cupping, moxibustion, Qi Gong, and Tai Qi. 

In China, TCM is officially state-supported and institutionalized. Ninety-five percent of Chinese hospitals have a TCM department [4]. Integrative medicine (TCM and western medicine) is a distinct discipline born out of postcommunist China in an effort to “modernize” TCM. It can refer to using modern scientific methods to investigate TCM therapies or to combining TCM and western Medicine in clinical practice. The Chinese government has actively supported this integration through numerous campaigns and edicts over the past 60 years [5].

Medical training in China is also unique as 98% of physicians have some TCM training [6]. Similar to osteopathic doctors in the United States, graduates of “TCM medical schools” in China are fully trained and credentialed in western medicine and can pursue allopathic residency and fellowship. Prior surveys of Chinese physicians have found extremely high rates of acceptance of TCM, likely in part due to their training [6]. However, there have been no systematic studies of the perceptions of Chinese oncologists on TCM specifically.

Likewise, we know that use of TCM is very high among the Chinese population. In 1997, TCM represented about 40% of all health care delivered in China [7]. However, there is scarce literature on TCM use by cancer patients in China, and the prior studies focus exclusively on breast cancer patients [8–10]. These studies have shown that 97% of breast cancer patients in China use CAM therapies [9, 10]. This is the first study to assess the use of TCM by a broader cancer population in China. This study is also unique in its parallel design of surveys of both physicians and patients at the same institution. 

Given China's rapid economic development and the continuous infusion of advanced western medicine therapies into China, how and why traditional medicine continues to be used and integrated with western therapies is an important question. Understanding which particular TCM therapies are being utilized, at what point during the care trajectory, and for what aims could inform clinical care and research in the West. In addition, Chinese immigrants to western countries are particularly likely to use TCM [11–15]. For clinicians caring for Chinese immigrants, it is important to understand culturally bound medical systems and to be aware of alternative therapies that their patients may be using for better counseling and coordination of care.

To characterize the utilization of and attitudes towards TCM among Chinese cancer patients, we conducted a survey of patients at a tertiary cancer center in Shanghai, China. We conducted a parallel survey of Chinese oncologists of estimates of patient TCM usage and opinions on risks and benefits of TCM in cancer patients. 

## 2. Subjects and Methods

Patients presenting to outpatient clinics at the Fudan University Shanghai Cancer Center (FUSCC) in Shanghai, China in June 2005 were screened to participate in this study. Patients were recruited from the central clinic waiting room. Eligible patients had to be over age 18, with a current or past diagnosis of cancer, able to speak and understand Mandarin Chinese, and presenting for a follow-up appointment with a FUSCC physician. An investigator assisted each patient with the survey in order to ensure facilitate comprehension. Physician surveys were distributed during faculty meetings or provided to department chairs for distribution. Patients signed a written informed consent, whereas physicians passively consented by returning the survey. The study was approved by the institutional review boards at both MD Anderson Cancer Center and FUSCC.

### 2.1. Questionnaire

The patient survey assessed use, motivation, provider, source of information, and physician notification for individual TCM therapies. Only patients that had used a particular therapy answered subsequent questions related to that therapy. Patients who had informed a physician of TCM therapy use were asked about physician response and those who had not were asked why use had not been discussed. All patients completed questions on demographic, treatment, and disease-related characteristics. Physicians were surveyed on estimated patient TCM use and motivation, as well as their own opinions on utility, concerns, and personal use of TCM.

The surveys were developed by a bilingual American researcher (JM) with two bilingual Chinese research nurses. Surveys were based on previous CAM surveys of cancer patients and physicians [16–19]. The surveys were developed in English, translated into Chinese, and then back-translated into English for verification. During instrument development, several items were altered based on input from the Chinese researchers regarding cultural appropriateness of item wording. The surveys were piloted on 10 outpatients and 5 physicians and questions were revised based on feedback from the patients and physicians. 

TCM therapies were classified into four major categories: (1) Chinese herbal medicine (CHM), including herbal decoctions, patent medicines, and herbal injections/infusions; (2) tonics/supplements and food therapy; (3) movement therapies; including Qi Gong and Tai Qi, and (4) acupuncture and moxibustion. Patients were also asked to “write-in” any additional CAM modalities used. 

### 2.2. Statistical Analysis

Participants were classified as TCM users if they used at least one therapy for any length of time in any of the four TCM categories. Chi-square tests were used to test the association between the use of TCM with each independent demographic and medical variable. Descriptive statistics were calculated for views among patients and physicians, and chi-square tests of independence determined if views differed using an alpha level of ≤0.05 as the cutoff for statistical significance. Two-sided tests were conducted.

## 3. Results

### 3.1. Patient Survey

#### 3.1.1. Patient Demographics

A total of 487 patients were screened for eligibility, and 352 (72.3%) were eligible for participation. Reasons for ineligibility included no cancer diagnosis (*n* = 69), new patient visit (*n* = 30), benign tumor (*n* = 7), being younger than 18 years old (*n* = 4), and unable to speak Mandarin (*n* = 25). 

Of the 352 eligible patients, 299 (82.2%) consented to participate and completed the survey. A total of 9 patients consented to participate but were later excluded because they did not take the survey (*n* = 1), withdrew (*n* = 3), or were ineligible (*n* = 5). Data from an additional 42 patients presenting to the TCM clinic for herbal decoctions were excluded, as these patients had 100% use of herbal decoctions and their inclusion would have skewed the data; thus, the final sample consisted of 248 patients. 

Basic demographic information is provided in Table 1. Ninety-nine percent were Han Chinese. Forty-eight percent came from Shanghai while the remaining patients represented 13 other provinces. Most patients had local disease (66%) that had not recurred (86%). Patients were evenly divided between being on and off treatment (49.4% and 50.6%, resp.) and represented 22 disease site diagnoses. The five most common cancer sites were breast (31.4%), GI (22.9%), lymphoma (11.7%), head and neck (10.8%), and lung (9.9%) (see Table 1). 

#### 3.1.2. Frequency of Use and TCM Use Predictors

Eighty-three percent of patients had used TCM since diagnosis. Use was greatest for tonics and food therapy (69.2%) and Chinese Herbal Medicine (CHM) (55.8%) (see Table 2). Only 1.3% (*n* = 3) of patients had used acupuncture and 6.8% (*n* = 8) had used movement therapies (Qi Gong or Tai Qi) (Table 2). Other less common TCM modalities used were Tui Na (*n* = 3) and Cupping (*n* = 1). Use of other CAM modalities was very rare and included yoga (*n* = 3), megavitamins (*n* = 1), music therapy (*n* = 1), prayer (*n* = 1), protein powder (*n* = 5), shark cartilage (*n* = 1), and tomato extract (*n* = 1). The most frequently used individual herbs, tonics, and food therapies were *Ling Zhi *(*Ganoderma lucidum*) (*n* = 94), *Hong Zao* (*Fructus Zizyphi jujubae*) (*n* = 27), *Xi Yang Shen* (*Panax quinquefolius*) (*n* = 21), and *Dong Chong Xia Cao *(*Cordyceps sinensis*) (*n* = 16).

Comparisons by chi-square analyses suggested that TCM use was associated with higher educational status (odds ratio = 2.6, *P* = 0.012) and higher income (odds ratio = 2.1, *P* = 0.042). Patients whose disease had not metastasized were more likely to use CHM (odds ratio = 1.9, *P* = 0.023) and tonics and food therapy (odds ratio = 2.1, *P* = 0.018). Females were more likely to use CHM (odds ratio = 1.9, *P* = 0.014), but use was otherwise similar between genders. TCM users and nonusers did not differ with respect to age, disease site, being on treatment, or disease recurrence (see Table 1).

#### 3.1.3. Communication of CAM Use with Physician

Overall, 63.5% of patients reported discussing their use of TCM with their physicians, but the rate of disclosure varied among therapies with highest rates for acupuncture (100%) and CHM (71%) and lowest for food therapy (28%) and Qi Gong (13%). The most common reason for not discussing TCM use with physicians overall was that physicians had never asked about use, although for food therapy and Qi Gong the most common reason was “not important for the doctor to know about use.” Very few patients were concerned that their doctor would discourage use (<11%). Most patients reported that their physician's response to disclosure was to encourage them to continue. The exception was for tonics/supplements for which more patients reported a neutral response. For all therapies, very few patients reported that their doctors asked them to stop using, with the highest rate for tonics/supplements (15.7%) (Table 2). 

#### 3.1.4. Provider and Information Source

Most patients (87%) received CHM from TCM doctors. However many patients reported allopathic physicians as their provider for herbal patent medicines and injections (35% and 67%, resp.), and 91.7% of allopathic physicians reported prescribing CHM to their patients, albeit to a minority. Most patients (71%) procured tonics and supplements from herbal pharmacists, while the remainder received these as gifts from friends and family. 

Most patients (74%) relied on friends/family/other patients for information on TCM. Overall, 33% of patients reported receiving information from their physician, but this rate varied greatly among therapies from 67% for herbal injections and 44% for CHM to 2% for tonics and 6% for movement therapies. Utilizing a physician as an information source was higher among females (*P* = 0.016). 

#### 3.1.5. Patient Reason for Use

Most patients used TCM to improve immune function (tonics and food therapy 72.7%, CHM 53.3%, Qi Gong 62.5%, acupuncture 33.3%) (Table 3). Many patients reported using biologically based TCM therapies for symptom relief and quality of life (CHM 51.8%, tonics and food therapy 50.6%). A majority of patients also reported using TCM treatments to cure or treat cancer or prolong life. This was the most common reason for using CHM (66.4%). Patients were most likely to use acupuncture for health problems other than cancer (66.6%). 

### 3.2. Physician Survey

#### 3.2.1. Physician Demographics

Of the 90 physician surveys distributed, 72 were returned and analyzed (80%). Physician mean age was 34 with 61% male. Fifty-four percent had been practicing for less than ten years. Fifty-three percent were surgical oncologists, 26% radiation oncologists, and 21% medical oncologists, which is representative of the distribution of employed physicians.

#### 3.2.2. Physician Estimate of Patient Use

A majority of physicians correctly estimated the percentage of patients using herbal injections and acupuncture (Table 4). Physicians tended to underestimate patient use of herbal decoctions, tonics/supplements, food therapy, and Qi Gong. Physicians overestimated patients' use of herbal patent medicine. Overall, the number of physicians recommending use of TCM was highest for herbal decoctions and herbal patent medicine (Table 3). Physicians with more years of practice were more likely to recommend herbal decoctions (*P* = 0.023) and Qi Gong (*P* = 0.05).

#### 3.2.3. Physician Belief in Utility

Physicians had very high rates of belief in utility of TCM treatments, particularly for herbal medicine. Fifty-seven percent of physicians indicated that CHM was useful in curing or treating cancer or prolonging life. Physicians were even more likely than patients to believe that biological therapies were useful for symptom relief and quality of life (CHM 87.5%, tonics and food therapy 76.4%). More experienced physicians were more likely to believe Qi Gong to be useful for treating cancer (*P* = 0.054) and older physicians were more likely to believe it is useful for improving immune function (*P* = 0.012). Physician's perception of patients' reason for use generally reflected their own belief in the utility of a particular therapy (Table 3).

#### 3.2.4. Physician Concerns

Physicians were most concerned with interference with treatment, lack of scientific evidence, and safety. Physicians had more concerns with herbs in general and with treatment interference specifically (40.3%) but were more likely to find herbs acceptable to use while on treatment (62.5%) than acupuncture (48.4%) or Qi Gong (33.3%) (Table 5). Physicians with more years of practice were more likely to believe herbs acceptable to use while on treatment (*P* = 0.012). Many physicians reported concerns with Qi Gong interference with treatment (35%). Surgical oncologists were much less likely to find Qi Gong acceptable to use while on treatment (*P* < 0.01). A significant portion of physicians were concerned with the safety of acupuncture (31%), but older physicians were less likely to be concerned (*P* < 0.02) (Table 5). 

#### 3.2.5. Physician Use of TCM

The majority of physicians (87.5%) had themselves used some form of TCM. Sixty percent had used three or more TCM therapies. Use was highest for CHM (82%) and lowest for Qi Gong (14%) and acupuncture (34%). More experienced and older physicians were more likely to have used acupuncture (*P* < 0.01 and *P* < 0.02, resp.).

## 4. Discussion

This study of 245 patients and 72 physicians at a Chinese cancer hospital is the first to systematically assess and compare Chinese physician and patient attitudes towards TCM. The importance of these results is threefold: first, to reflect a unique medical system and demonstrate the effect of culture, history, and politics on utilization of and attitudes towards CAM; second, to elucidate what and why particular TCM therapies are being utilized which could inform clinical care and research in the West; third, to increase cultural competence of western practitioners caring for Chinese immigrants who commonly use TCM.

The use of TCM by Chinese cancer patients is exceptionally high, and physicians are generally well informed and supportive of their patients' TCM use. The 83.5% rate of TCM use by patients in our study is similar to that found in the few prior studies of Chinese cancer populations [9, 10] and significantly higher than CAM usage reported in most western countries [2, 3, 20]. This is not surprising given that TCM is intricately intertwined with the history, culture, and politics of China. Additionally, TCM is institutionalized in China and actively promoted by the Chinese government as an area of research, a point of pride, and even as a commodity for export.

As in other populations, Chinese users of CAM tended to be more educated and in higher income brackets [2]. Given that these therapies are “traditional medicine” in China, relatively inexpensive, and often covered by health insurance, it was somewhat surprising that the demographics would be parallel to those in western countries. 

In this study, the therapies most commonly used were various forms of Chinese herbal medicines, tonics and food therapies. This is similar to many other countries where biologically based therapies are the most commonly used CAM [20–22]. However, this is in stark contrast to the focus of research into TCM in the West where acupuncture is the dominant TCM therapy.

Surprisingly, cancer patients in China very rarely use acupuncture, and the few who do use it do so for health problems other than cancer. The majority of physicians surveyed believe that acupuncture is useful for symptom relief and improving QOL, but rarely recommended acupuncture to their patients. Numerous studies have shown acupuncture to be a useful adjunct to relieving cancer and treatment-related symptoms [23–55]. Most of this research has been performed in the West, while most research on TCM cancer care in China is focused on herbals. At FUSCC, there are 14 herbalists but only two acupuncturists. Many physicians had concerns about the safety of acupuncture. Based on informal follow-up questioning, it seems this concern stems from a belief that acupuncture may seed metastases. There has only been one case report in the literature of a speculated case of acupuncture spreading cutaneous metastases when an acupuncture needle was directly inserted into a malignant lymph node, but, again, even this case was speculative [56]. It is contraindicated to insert acupuncture needles into a tumor due to this concern for seeding potential and it is similarly contraindicated to puncture infected skin. Overall, however, acupuncture is a safe, minimally invasive procedure with very few side effects and few absolute contraindications [57–62]. 

Many physicians also expressed concern that Qi Gong may interfere with conventional treatment and felt that Qi Gong was the least acceptable for patients to use while on treatment compared to all TCM therapies. This was surprising given that Qi Gong is purely a movement-based mind-body practice that falls in the CAM category of an “energy” manipulation and has none of the potential interactions with chemotherapy of herbal treatments and is not an invasive procedure such as acupuncture. At the same time, the majority of physicians also indicated they thought Qi Gong would help improve QOL and they did not indicate any safety concerns. Although we did not probe as to the specific reasoning behind the concerns, physicians could be concerned that the time involved with Qi Gong practice could detract from getting conventional treatment or that patients forego conventional treatment in favor of Qi Gong. During the time of this survey, our group was also conducting a study of Qi Gong and quality of life in breast cancer patients undergoing radiotherapy which encouraged daily practice. This may have impacted physician attitudes towards Qi Gong. Additionally, since the Falun Gong controversy of the 1990s, there has been lingering skepticism towards Qi Gong of which Falun Gong is an outgrowth decried as a cult by the Chinese government [63]. 

In contrast to many other populations, most Chinese patients discuss use of TCM with their oncologists and find their physicians to be generally supportive. In fact, over 90% of physicians reported having prescribed TCM to their patients and many physicians use TCM themselves. This is likely a reflection of the fact that almost all western trained physicians in China have had some TCM training [4]. 

The majority of Chinese physicians believe that TCM therapies have utility in cancer care. Unlike their western colleagues, many Chinese oncologists believe these complementary therapies useful not only for symptom control but also for treating cancer (particularly CHM). However, many physicians also report concern about the lack of evidence supporting CHM. Not only is this concern discrepant with their reported personal beliefs in the utility of CHM but also at odds with the vast amount of positive studies published in Chinese medical journals. This incongruity may be a reflection of the knowledge that the quality of the RCTs on TCM published in Chinese medical journals, while improving, remains quite poor [64]. There also seems to be an *a priori* belief that TCM is useful and beneficial given the historical experience, and the role of scientific research is to prove to the world the value of this cultural legacy while at the same time using scientific principles to systematize and modernize TCM. Indeed, this view is reflected in Communist party slogans to “inherit and carry forward the treasure-house of TCM [5].” 

As to why Chinese patients use TCM, many patients report using for symptom control or as an “immune booster,” but the most common reported reason for many of the biologics was to treat or cure cancer or to prolong life. In clinical practice, purified or semipurified herbal extracts made into injections or infusions are often used as chemotherapeutics, and research into these extracts is appropriately focused on botanical as novel drug. However, herbal decoctions, the true “traditional” Chinese medicine and the form most often used, are complex whole herb extractions with basic formulas made up of 4–10 individual herbs with additional herbs added or subtracted based on patient symptomatology. Patients visit an herbalist bimonthly for years for prescriptions for individualized formulas of herbs which they then decoct at home. Based on the large amount of write-in answers, the full perceived utility of these decoctions may not have been well captured in our survey which was adapted from US-based surveys of CAM use. Many write-in answers reflected TCM-specific concepts such as to tonify Qi, yin, or blood or the restoration of balance. In speaking to physicians, these functions of Chinese herbal medicine are thought to be important to both consolidate the effects of western treatments as well as to help the body recover from these treatments. These functions are reflected in the oft-heard sayings, “Chinese medicine treats the root, while western medicine treats the manifestations” or “Chinese medicine treats chronic; western medicine treats acute” [5]. This possibility of Chinese medicine as a “consolidative” or maintenance therapy is an intriguing area for future research given that it would minimize potential for herb-drug interactions as patients would be “off-treatment” and is a therapy phase currently lacking in the western treatment of many cancers. 

Several limitations of this study must be acknowledged. The findings may not be broadly representative of cancer patients in China as it was conducted at one tertiary cancer center in a large relatively affluent urban center, although most patients came from outside of Shanghai and the low income and education levels of the sample are reflective of the population as a whole. The physicians in this survey tended to be young with less than 10 years experience and as demographics were not collected on nonresponders it is unknown if this could represent respondent bias. However, the physician response rate was very high at 80%. The surveys were developed based on prior surveys conducted in the US and may therefore not have reflected Chinese values. We attempted to minimize this cultural bias through the engagement of Chinese colleagues in adapting the instruments and then piloting and refining the instruments. The categorization of the biologics was developed in collaboration with Chinese colleagues but is not standardized and patients may have interpreted these categories differently.

This first systematic assessment of Chinese physician and patient attitudes towards TCM in cancer care demonstrated high use and acceptance and general concordance between patients and physicians. The response rates for both patients and physicians were unusually high, suggesting the findings are representative of the population being assessed. The high rate of use of TCM is a reflection of a uniquely integrated medical system with a long history; however, the findings have relevance to practitioners in the West, particularly to clinicians caring for Chinese immigrants, TCM practitioners, and CAM researchers. 

Ideally, more preclinical and clinical research should be focused on the form of TCM most commonly used by the Chinese in cancer care, Chinese herbal decoctions. However, there are significant barriers to this type of research given that decoctions involve whole herbs with many active and inactive ingredients and impurities greatly impacting quality control [65] and formulations are individualized and change over time in response to patients' changing clinical patterns. Areas for future qualitative research include patient satisfaction and quality of life and more in-depth questions regarding duration and patterns of use, further questioning of physicians on the discordance between their beliefs in utility and personal use and their concerns regarding lack of evidence, and better understanding the concerns with acupuncture and Qi Gong in cancer care. Education on the true risks and benefits of different TCM therapies may result in improved symptom control for this population of patients.

## Figures and Tables

**Table 1:** Use of TCM by demographic, disease, and treatment-related variables.

Variable	All respondents (*n* = 248) (%)	TCM users (*n* = 207) (%)	Nonusers (*n* = 41) (%)	*χ* ^2^	*P*
Age					
≤51 years	47.8	41.6	36.6	2.46	0.117
>51 years	52.4	41.6	63.4		
Gender					
Male	40.3	38.0	53.7	3.49	0.062
Female	59.7	62.0	46.3		
Education					
≤Middle school	57.9	54.2	75.6	6.38	0.012
>Middle school	42.1	45.8	24.4		
Annual Income					
≤1250 USD	35.3	32.0	50.0	4.25	0.039
>1250 USD	64.7	68.0	50.0		
Metastasized					
Yes	34.3	36.5	24.4	2.24	0.135
No	65.7	63.5	75.6		
Recurred					
Yes	14.3	14.2	14.6	0.01	0.935
No	85.7	85.9	85.4		
On treatment					
Yes	49.4	50.5	43.6	.624	0.430
No	50.6	49.5	56.4		
Diagnosis					
Breast	31.4	35.3	12.8	10.4	0.065
GI	22.9	20.1	35.6		
Head and neck	10.8	10.9	10.3		
Lung	9.9	8.7	15.4		
Lymphoma	11.7	12.0	10.3		
Other	13.5	13.0	15.4		

**Table 2:** Proportion of patients who used TCM and discussed TCM with physicians, reason why use is not discussed, and physician response to disclosure.

			Reason why use is not discussed			Physician response to use disclosure		
Therapy	Used (%)	Discussed with physician (%)	Doctor never asked	Doctor would discourage	Not important for doctor to know	Encouraged me to continue	Asked me to stop	Neutral
			% yes	% yes	% yes	% yes	% yes	% yes
TCM overall	83.5	63.5						
Chinese herbal medicine (CHM)	54.5	70.9						
Herbal decoctions	47.6	65.2	65.0	7.5	17.5	54.1	5.4	36.5
Patent medicines	31.8	68.8	52.2	8.7	30.4	71.7	5.7	20.8
Herbal injections	2.5	100.0	N/A	N/A	N/A	83.3	0.0	0.0
Tonics and food therapy	69.2	40.5						
Tonics/supplements	50.4	41.5	63.8	10.1	11.6	33.3	15.7	49.0
Food therapy	41.5	27.9	44.1	0.0	51.5	70.4	7.4	14.8
Movement/physical therapies	6.8	23.1						
Qi Gong	3.4	12.5	42.9	0.0	57.1	100.0	0.0	0.0
Tai Qi	4.0	22.2	71.4	0.0	28.6	100.0	0.0	0.0
Acupuncture	1.3	100.0	N/A	N/A	N/A	100.0	0.0	0.0

**Table 3:** Reasons for TCM use: patients' response versus physicians' perceptions and physician beliefs about TCM utility.

Reason	Patient reason for use* % yes	Physician perception of patient reason for use % yes	*χ* ^2^	*P*	Physician belief about utility % yes
*Chinese herbal medicine *(*CHM*)					
Cure or treat cancer or prolong life	66.4	57.0	1.82	0.177	54.2
Symptom relief or improve QOL	51.8	83.3	20.0	<0.001	87.5
Improve immune function	53.3	86.1	22.2	<0.001	83.3
Health problems other than cancer	4.4	40.3	43.6	<0.001	37.5
*Tonics and food therapy *					
Cure or treat cancer or prolong life	43.0	36.1	1.00	0.317	26.4
Symptom relief or improve QOL	50.6	75.0	12.4	<0.001	76.4
Improve immune function	72.7	75.0	0.14	0.708	72.2
Health problems other than cancer	4.7	75.0	132	<0.001	20.8
Qi Gong*					
Cure or treat cancer or prolong life	50.0	22.2			13.9
Symptom relief or improve QOL	12.5	51.4			52.8
Improve immune function	62.5	43.1			38.9
Health problems other than cancer	12.5	12.5			9.7
Acupuncture*					
Cure or treat cancer or prolong life	33.3	20.8			18.1
Symptom relief or improve QOL	0.0	66.7			68.1
Improve immune function	33.3	29.2			27.8
Health problems other than cancer	66.7	26.4			27.8

*The total number of patients using Qi Gong was only 8 and using acupuncture was only 3, thus comparative statistics were not conducted.

**Table 4:** Percent of patient use, physician estimate of patient use, and patients asking about therapy, patients to whom physicians recommended therapy.

Therapy (% range of physicians)	Patient use (%)	Physician estimate of patients using this therapy (%)	Patients asking about this therapy (%)	Patients to whom physician has recommended use of this therapy (%)
TCM overall	83.5			
Herbal decoctions	47.6			
0%		9.9	4.2	12.7
1–25%		39.4*	33.8	**45.1**
26–50%		33.8	18.3	26.8
>50%		16.9	**43.7**	15.5
Patent medicine	31.8			
0%		6.9	1.4	4.3
1–25%		23.6	33.8	**44.3**
26–50%		16.7	26.8	30.0
>50%		**51.9**	**38.0**	21.4
Injections	2.5			
0%		15.3	18.1	37.5
1–25%		**73.6**	**66.7**	**50.0**
26–50%		5.6	9.7	6.9
>50%		5.6	5.6	5.6
Tonics/supplements	50.4			
0%		14.5	5.7	**44.3**
1–25%		**44.1**	28.6	**44.3**
26–50%		18.8	25.7	5.7
>50%		23.2	**40.0**	5.7
Food therapy	41.5			
0%		20.6	17.1	31.3
1–25%		**42.7**	**34.3**	**38.8**
26–50%		16.2	27.1	13.4
>50%		20.6	21.4	16.4
Acupuncture	1.3			
0%		39.4	43.3	**47.8**
1–25%		**56.1**	**52.2**	43.3
26–50%		4.6	4.5	7.5
>50%		0.0	0.0	1.5
Qi Gong	3.4			
0%		**48.5**	**55.1**	**75.4**
1–25%		43.9	34.8	18.8
26–50%		4.6	7.3	2.9
>50%		3.0	2.9	2.9

**Table 5:** Physician concerns.

Therapy	Safety % yes	Unrealistic expectations % yes	Cost % yes	Interference % yes	Lack of evidence % yes	Acceptable to use while on treatment % yes
Chinese herbal medicine (CHM)	37.5	33.3	27.8	40.3	48.6	62.5
Tonics and food therapy	33.3	19.4	36.1	48.6	38.9	58.3
Qi Gong	13.9	5.6	2.8	34.7	26.4	33.3
Acupuncture	30.6	9.7	2.8	19.4	19.4	48.6
